# Infective Endocarditis Caused by *Abiotrophia defectiva*: A Unique Case Complicated by Mitral Valve Perforation

**DOI:** 10.1002/ccr3.70400

**Published:** 2025-04-06

**Authors:** Ra'ed Ababneh, Laith Rhabneh, Shahd Qaddour, Emad Algorani, Mohammed Aloqaily, Cheikh Ahmed Abool Maaly

**Affiliations:** ^1^ Internal Medicine Hamad Medical Corporation Doha Qatar; ^2^ Internal Medicine HMH Ocean University Medical Center USA; ^3^ Diagnostic Radiology King Abdullah University Hospital Irbid Jordan; ^4^ University of Maryland Medical Center Midtown Campus Baltimore Maryland USA; ^5^ Cardiology Hamad Medical Corporation Doha Qatar

**Keywords:** *Abiotrophia defectiva*, cardiovascular, dental procedure‐related infections, endocarditis complications, infective endocarditis

## Abstract

*Abiotrophia defectiva* , a nutritionally variant streptococcus, represents a rare yet serious cause of infective endocarditis (IE), accounting for 1%–2% of the total IE cases. It affects native valves in 10% of patients, and it is implicated in catastrophic complications. A 33‐year‐old medically free male presented with a persistent nonproductive cough for 4 months. It was associated with unintentional weight loss (10 kg); however, he denied any other symptoms. His symptoms started after he had a dental implant. Physical examination was unremarkable except for a systolic murmur. Upon further investigations, a blood culture showed *Abiotrophia defectiva* , and echocardiography demonstrated severe mitral regurgitation originating from the perforation site located on the posterior leaflet measuring 10 × 4 mm, in addition to 2 masses suggestive of vegetations, the largest measuring 10 × 6 mm. The patient was hospitalized as a case of IE and acute mitral valve regurgitation. In light of mitral valve perforation, large vegetation, and bacteremia, an urgent multidisciplinary decision to proceed with mitral valve replacement was made, and the patient received a total of 6 weeks of antibiotics. Physicians should remain highly vigilant for IE, its rare causes, and associated complications. In addition, managing complex cases of IE necessitates a multidisciplinary team approach between cardiology, infectious diseases, and cardiac surgery teams.

**Summary**

-
*Abiotrophia defectiva* infective endocarditis is a rare, challenging diagnosis often linked to recent dental procedures.
- Its complications, including mitral valve perforation, necessitate early recognition, targeted antibiotics, and timely surgical intervention.

**Abbreviations**
AHA: American Heart Association
CRP: C‐reactive protein
CTICU: Cardiothoracic Intensive Care Unit
IE: infective endocarditis
MPG: mean pressure gradient
NVS: nutritionally variant streptococcus
TEE: transesophageal echocardiogram
TTE: transthoracic echocardiogram
WBC: white blood cell count

## 1 Introduction

*Abiotrophia defectiva* is a nutritionally variant streptococcus, accounting for 1%–2% of infective endocarditis (IE) cases [1]. Despite being a commensal organism in the oral, urogenital, and gastrointestinal tracts, it can cause severe infections such as bacteremia, brain abscess, meningitis, and osteomyelitis [2]. Its fastidious nature and strict nutritional requirements frequently lead to blood culture‐negative IE, complicating diagnosis and treatment [2].

The name Abiotrophia, meaning “life nutrition deficiency,” reflects its need for supplemented media to grow [3]. *A. defectiva* is a nonmotile, gram‐positive, catalase‐negative coccus that is pyridoxine‐dependent and exhibits satellitism around Staphylococcus streaks. Automated blood culture systems are essential for its detection, as it can exhibit pleomorphic Gram stain morphology [4, 5].

*A. defectiva* ‐associated IE primarily affects individuals with preexisting valvular disease or prosthetic valves but can also occur in those without known risk factors. It is associated with complications like valvular destruction, septic embolization, and a high rate of treatment failure despite antibiotic sensitivity [6]. Septic embolization occurs in up to one‐third of cases, and relapse rates reach 17% [7]. Prosthetic valve replacement is required in 27% of cases, and mortality is slightly higher than with IE caused by viridans streptococci or enterococci [8].

A review of the literature reveals several reported cases of *A. defectiva* IE in patients without prior heart disease, including:

- A 58‐year‐old woman developed *A. defectiva* endocarditis after a root canal procedure. She had no significant medical history, and the infection led to severe complications, including heart valve destruction and systemic embolization [9].
- An 8‐year‐old girl, previously healthy, presented with fever and right‐sided limb weakness. She was diagnosed with *A. defectiva* IE complicated by embolic events leading to neurological deficits [10].
- A previously healthy adult male was diagnosed with *A. defectiva* IE, which resulted in multiple embolic events, including splenic and renal infarctions, necessitating surgical intervention [11].

What differentiates this case is the severe mitral regurgitation and valve perforation, which necessitated surgery and prevented further systemic embolization.

## 2 Case Presentation

A 33‐year‐old male with no significant past medical history presented to the emergency department with a 4‐month history of persistent dry cough, unintentional weight loss of 10 kg, and reduced appetite. He had no fever, chills, chest pain, or shortness of breath. His symptoms started following a dental implant procedure approximately 4 months prior to their emergence. A new systolic murmur was noted on physical examination, which led to further investigations.

Laboratory investigations revealed leukocytosis (WBC 14.4 × 10^9^/L), mild anemia (Hb 11.9 g/dL), thrombocytosis (platelet count 475 × 10^9^/L), and an elevated CRP (69 mg/L), indicative of systemic inflammation. Procalcitonin was within normal limits (0.09 ng/mL).

**FIGURE 1:**
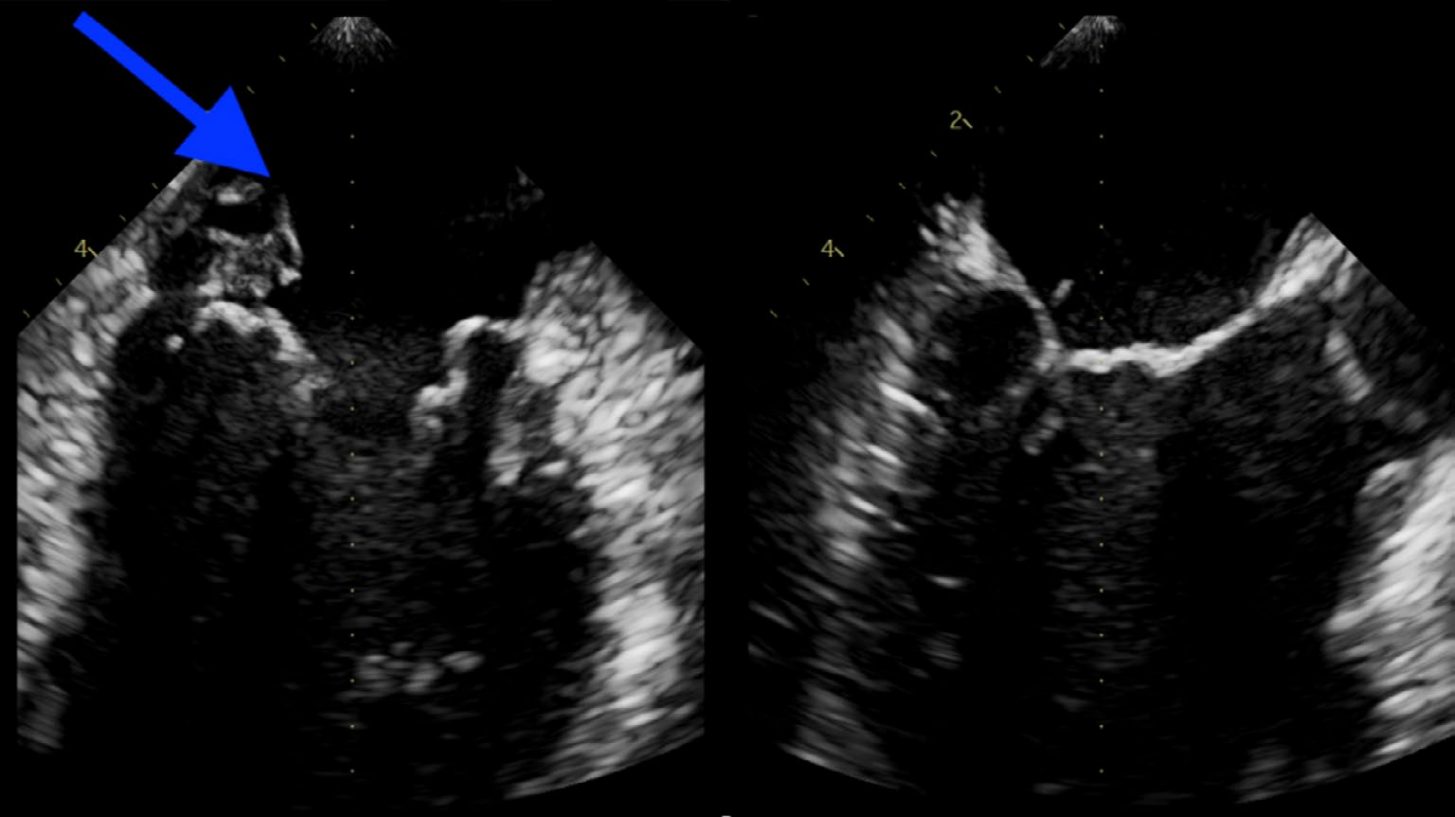
TTE showing perforated posterior mitral leaflet.

**FIGURE 2:**
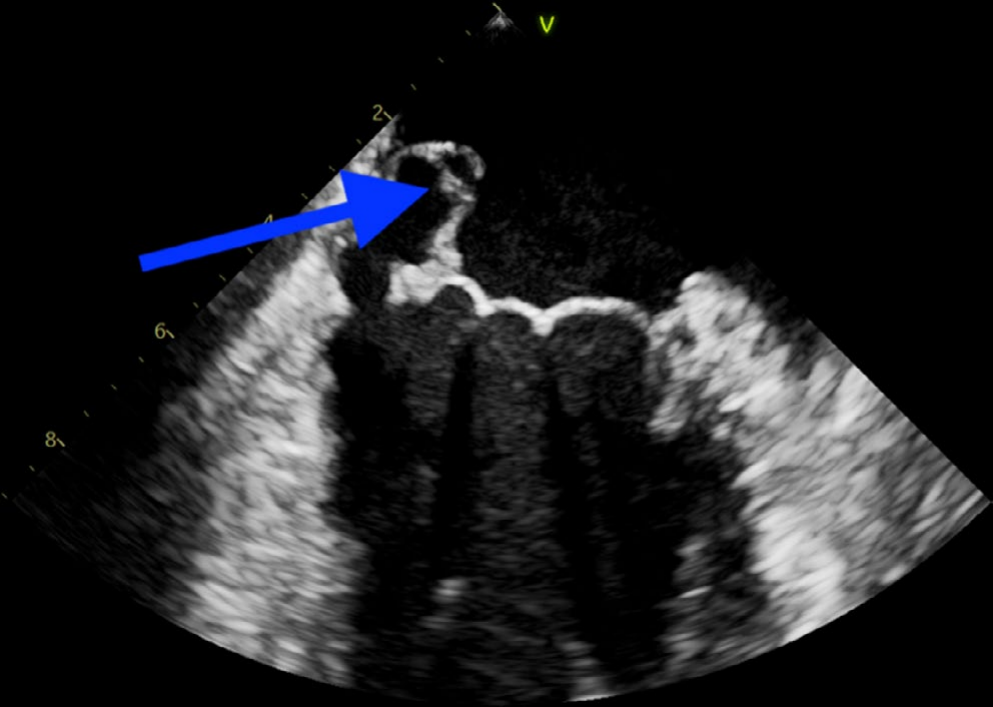
TTE demonstrating large mitral vegetations over the posterior mitral leaflet.

**FIGURE 3:**
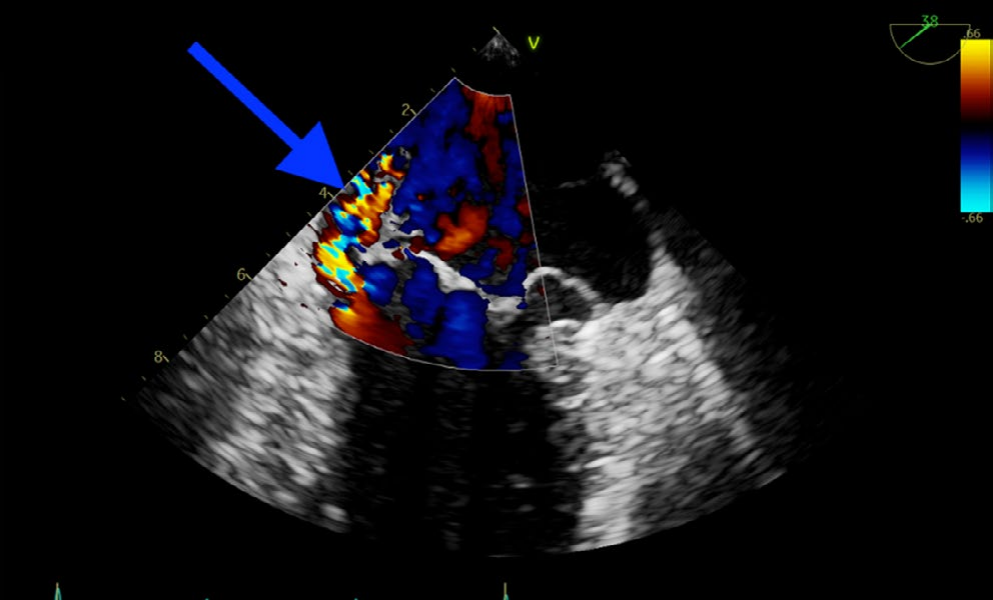
TTE with Doppler showing significant mitral regurgitation due to posterior mitral valve leaflet perforation.

Transthoracic echocardiogram (TTE) revealed two large vegetations attached to the mitral valve, along with perforation of the posterior mitral leaflet (Figures 1 and 2), causing significant mitral regurgitation (Figure 3). Additionally, blood cultures grew *A. defectiva* , a gram‐positive bacterium typically associated with infective endocarditis following dental procedures.

Moreover, a Transesophageal echocardiogram (TEE) demonstrated a severe eccentric mitral regurgitation secondary to perforation of the posterior mitral leaflet (P3), with possible malcoaptation between the A1 and P1 leaflets. A large, highly mobile vegetation was attached to the atrial side of the P3/A3 segment of the mitral valve.

The patient's persistent symptoms, combined with the findings of mitral valve perforation, raised the concern for IE complicated by acute mitral regurgitation. A decision was made to proceed with mitral valve replacement due to the risk of worsening valvular dysfunction, embolism, and the need for long‐term antibiotic therapy.

### 2.1 Management and Surgical Intervention

The patient was empirically started on intravenous ceftriaxone following the preliminary identification of gram‐positive cocci in pairs and chains in the blood culture. After identification of the causative organism, antibiotic therapy was switched to penicillin G and gentamicin based on expert opinion. Subsequently, the patient developed leukopenia, with a white blood cell (WBC) count dropping to 1800/mm^3^. Given the suspicion of penicillin being the culprit, the antibiotic regimen was adjusted to ceftriaxone and gentamicin. Of note, WBC count improved following the discontinuation of penicillin.

A shared decision to proceed with surgical intervention was made, and the patient underwent mitral valve replacement with a 29 mm St. Jude mechanical mitral valve. The surgical procedure was performed successfully without complications.

Intraoperative findings were noticeable for severe eccentric mitral regurgitation due to perforation of the posterior mitral leaflet, destruction of segments A3 and P3, along with the adjacent commissure, and evidence of fresh infection extending to the annulus. Subsequently, the large, highly mobile vegetations attached to the atrial side of the posterior leaflet were removed, and the mitral valve was replaced with a mechanical valve.

Importantly, the procedure was uneventful, and the patient came off the bypass in sinus rhythm, without the need for inotropic support. Postoperative TEE revealed a well‐seated mechanical valve without regurgitation or paravalvular leak. The mean pressure gradient across the new valve was 2.5 mmHg, indicating excellent valve function.

### 2.2 Postoperative Course

The patient had a smooth postoperative recovery and was transferred to the Cardiothoracic Intensive Care Unit for monitoring. Sedatives were gradually tapered off, and the patient was extubated.

Given the intraoperative findings and concerns for ongoing infection, the Infectious Disease team was consulted postoperatively, tissue cultures were obtained, and ceftriaxone was re‐started on the day of the surgery. It is worth mentioning that tissue cultures from four intraoperative samples did not show microbial growth. Based on these results and the patient's status, the final shared decision was to continue ceftriaxone for another two weeks. Fortunately, the patient remained stable throughout his hospital course and was later discharged in good condition.

### 2.3 Follow‐Up

The patient resumed daily activities without limitations and continued regular follow‐ups in both cardiology and infectious disease outpatient clinics without new complaints or complications.

## 3 Discussion

This case describes infective endocarditis (IE) caused by an uncommon bacterial pathogen, *Abiotrophia defectiva* . This organism is known to infect defective heart valves and immunocompromised patients. However, previously reported cases have highlighted its rare occurrence in immunocompetent individuals with structurally normal heart valves [10, 11, 12]. From the limited cases described in the literature, a high prevalence of neurological complications has been reported [12].

This case is unique as it documents *A. defectiva* IE in a patient without preexisting valvular abnormalities or immunosuppression. Additionally, it is the first to describe valvular perforation as a sequela of *A. defectiva* IE in a patient without preexisting valvular abnormalities or immunosuppression.

Recent dental procedures have been identified as a predisposing factor for *A. defectiva* IE, as oral colonization of this organism has been reported in 11.8% of healthy individuals [13]. This underscores the importance of recognizing dental and mucosal procedures as potential triggers, particularly in patients presenting with nonspecific symptoms and systemic infection. Furthermore, *A. defectiva* IE has also been well‐documented in the context of sepsis, highlighting its potential severity [6].

Treatment regimens for *A. defectiva* IE are categorized based on penicillin sensitivity [4]. In this case, *A. defectiva* was susceptible to penicillin, allowing for a more flexible treatment regimen. Unfortunately, the patient developed a drop in WBC count, necessitating a change in antimicrobials, and was finally treated with daily ceftriaxone for at least four weeks, as recommended by the American Heart Association (AHA) [4].

This case highlights the critical role of early diagnosis and management by a multidisciplinary team in improving patient outcomes. The patient's severe mitral regurgitation and valve perforation necessitated urgent surgical valve replacement to prevent complications such as embolic events and heart failure, which are leading causes of mortality associated with *A. defectiva* IE [8].

Ultimately, this case reinforces the importance of considering *A. defectiva* as a potential causative pathogen in IE, particularly following dental or mucosal procedures. Early recognition, standardized antibiotic therapy, and timely surgical intervention remain essential for optimizing outcomes in these complex cases [14].

## 4 Conclusion

Infective endocarditis can present with nonspecific symptoms, making early diagnosis challenging. In this case, the combination of echocardiography, blood cultures, and clinical presentation led to the timely diagnosis of severe mitral regurgitation due to IE. Furthermore, a successful mitral valve replacement was crucial in managing the patient's severe valvular dysfunction, and he was discharged with a plan for continued antibiotic therapy and follow‐up care. This case emphasizes the importance of multidisciplinary teams enrollment and early surgical intervention in cases of infective endocarditis with significant valve destruction to achieve favorable outcomes.

## Author Contributions

**Ra'ed Ababneh:** writing – original draft. **Laith Rhabneh:** project administration, supervision, writing – original draft. **Shahd Qaddour:** investigation. **Emad Algorani:** writing – review and editing. **Mohammed Aloqaily:** project administration. **Cheikh Ahmed Abool Maaly:** supervision.

## Ethics Statement

The authors have nothing to report.

## Consent

Written informed consent was obtained from the patient for the publication of his clinical details and any accompanying images in this case report.

## Conflicts of Interest

The authors declare no conflicts of interest.

## Data Availability

The authors have nothing to report.
